# Transparent Displays Utilizing Nanopatterned Quantum Dot Films

**DOI:** 10.1038/s41598-018-20869-1

**Published:** 2018-02-06

**Authors:** Sang-ho Shin, Boyeon Hwang, Zhi-Jun Zhao, So Hee Jeon, JooYun Jung, Ji-Hye Lee, Byeong-Kwon Ju, Jun-Ho Jeong

**Affiliations:** 1 Department of Electrical Engineering, College of Engineering, Korea University, Seoul, 02841 Korea; 2 Department of Nano Manufacturing Technology, Korea Institute of Machinery and Materials (KIMM), Daejeon, 34103 Korea

## Abstract

We report the realization of a transparent display using glass covered by a nanopatterned quantum dot (QD) film with good transmittance. The film was fabricated by nanoimprint lithography (NIL) and spin coating of colloidal QDs with specificexcitation maxima. The produced nanopatterned QD film was attached to transparent glass, enabling active image generation using a laser light source of a specific wavelength. Selective light emission was induced by strongly exciting the laser-exposed film surface, creating desired images, with color modulationenabled by controlling the QD layer (dozens of nanometers in size) *via* nanopatterning. The nanopatterned QD film used for image generation exhibits excellent transmittance (>80%), and can be used for transparent displays, with image realization in both bright and dark spaces. The fabricated displays have wide viewing anglesowing to their good light emission characteristics, and the fabrication through spin coating renders the fabrication process simple and applicable to large areas.

## Introduction

The recently developed transparent displays find numerous applicationssuch as flexible displays that can be bent like paper, wearable displays^1,2^, and mirror displays^3^ that function as both mirror and screen at the same time. In these displays, the information is presented on a light-transmitting panel, creating an impression of images created on transparent glass^4,5^. Such displays can be divided into projection^3^ and transparent displays^6^. The former utilizes light projection on a transparent screen where an image needs to be displayed and includes head-up displays (HUDs)^7,8^ that project light on a screen, *e.g*., airplane or automobile windows, and head-mounted displays (HMDs)^3^ that project light directly on the human eye. In the past, HUDs were used for displaying various warplane informationnowadays, they are routinely used in automobile systems implementing augmented reality^9,10^. HMDs^11,12^ utilized in devices such as glasses or goggles project light directly on the human eye, making both the real-world image (viewed through the lenses) and the display image visible at the same time. Transparent displays function by changing the transmittance of the light-emitting display materials, and utilize either liquid crystals (LCDs)^13^ or organic light-emitting diodes (OLEDs)^14,15^. LCDs can hardly provide clear image quality outdoors, owing to their poor transmittance (~20%), which is caused by loss of light as it passes through the polarizing plate, color filter, and back light^16^. The development of light-emitting diodes (LED) technology inspired the realization of transparent OLED displays featuring incorporated organic semiconductors^17^, which are self-emissive and do not require a polarizing plate, color filter^18^, and back light, providing transmittances as high as 40%^19^. In particular, display characteristics of OLEDs, which are highly applicable in transparent displays, are still being studied. Heterostructured OLEDs based on glass substrates coated with transparent Indium Tin Oxide (ITO), which present a transparency of 70% in the visible light region, can be applied to high-resolution full-color displays or HUD display applications because the top electrode and electron injection layer are thin and transparent^20,21^.

The latest augmented reality displays utilize the transparent display technology to modify the front windows of airplanes, cars, and other means of transportation to display information such as the velocity and current location^22,23^. Three-dimensional augmented reality can be realized by complementing the real-world picture with informative images, which is currently achieved*via* HUDs that simultaneously display the outside view and the corresponding driving-related information. However, it is worth noting that the transparency of such displays is still far from satisfactory. LCDs and OLEDs feature transmissions of ~20% and ~40–50%, respectively, but objects behind the substrate are not seen clearly and distinctly. Moreover, because these devices are mostly used under bright outdoor illumination, a very bright light source is needed to offset the low (4%) reflectivity of glass used in projection displays, hindering practical applications.

In this study, a nanopatterned QD film^24,25^ was fabricated by nanoimprint lithography (NIL) and spin coating for use in transparent displays (*e.g*., HUDs)^26,27^. The fabricated film not only allows QD emission wavelength tuning by the choice of QD dimensions, but also modulates the emission intensity *via* nanopatterning. The existing QD thin films neither provide adjustable coating thickness nor exhibit good QD layer uniformity^28^. On the contrary, our nanopatterned QD film employs a nanoscale line pattern, with the QD layer coating thickness being effectively adjusted by varying the nanopattern depth^29,30^. Moreover, the nanopattern surface quality could be improved by the deposition of SiO_2_ layers and O_2_ plasma treatment^31^. Becausethe fabricated nanopatterned QD film has a negatively charged surface,uniform QD layer deposition could be achieved by spin coating^32^. Moreover, single-layer deposition enabled easy and effective color modulation. When exposed to red, green, and blue lasers in the visible light range, selective light emission due to QD excitation enabled custom image generation on the nanopatterned QD film surface, and clear images were realized under bright open-air conditions. The fabricated film exhibits 80% transmittance in the entire visible light range. Thus, when it was used in transparent displays, objects on the back side of the display screen were clearly visible, and distinct images could be projected on the screen^33^. As for fabrication, the simple NIL and spin coating processes are cost-effective, and the wide viewing angle realized by QD excitation enables the construction of large-area displays.

## Results and Discussion

**Figure 1:**
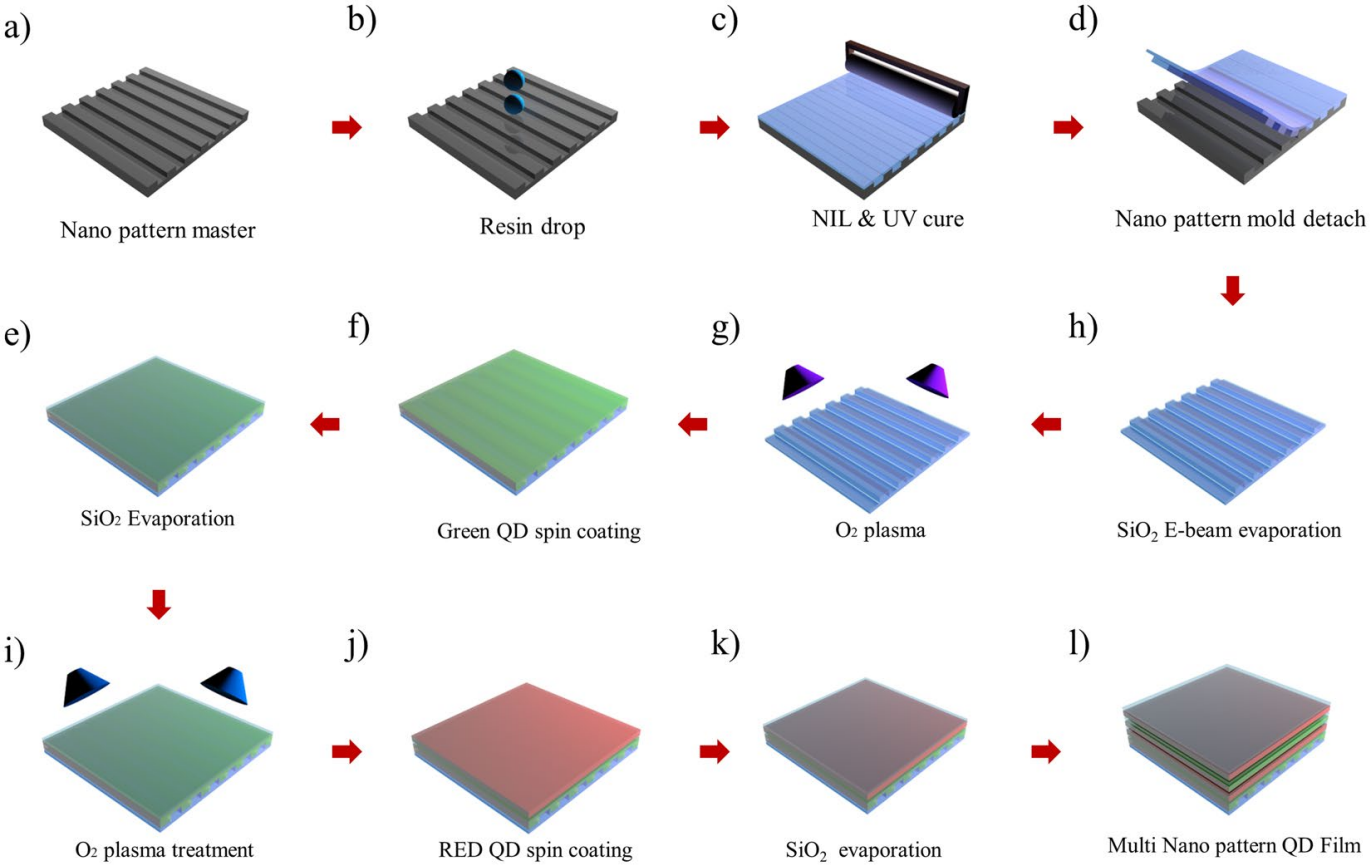
Schematic illustration of the nanopatterned QD film fabrication using nanoimprint lithography and spin coating. (**a**) Nano line pattern master, (**b**) an imprint resin drop on the nano line master, (**c**) UV curing of the imprinted resin, (**d**) separation of the master and the cured nanopatterned mold, (**e**) SiO_2_ e-beam evaporation on the nanopattern mold, (**f**) O_2_ plasma treatment of the SiO_2_ mold, (**g**) green QD spincoating on the O_2_ plasma-treated mold, (**h**) SiO_2_E-beam evaporation, (**i**) O_2_ plasma treatment on the SiO_2_/E-beam-evaporated QD, (**j**) spin coating of the red QD, (**k**) SiO_2_ evaporation, (**l**) finished multi-nanopatterned QD film.

As shown in Fig. 1, the nanopatterned QD film was fabricated by NIL using a QD suspension. To prepare the film, a nanoimprinting resin was dropped two or three times on the master with a pre-formed line pattern of nanosize width. Subsequently, the master was covered by a polyethylene terephthalate (PET) film, and the dropped resin was uniformly flattened using a roller. The resulting assembly was UV-cured for 90 s. The PET film was separated from the master and post-cured for 90 s, affording the nanopatterned film.

The master width pattern featured lines with a line width of 200 nm, pitch of 800 nm, and depth of 100 nm. When the nanopatterned film fabricated thus is duplicated, the resulting film exhibits a line width of 600 nm and pitch of 800 nm. In order to improve the adhesion between QDs and the film surface, a 10 nm buffering SiO_2_ layer was deposited at 1.0 Å/s using an E-beam evaporator. To fill the nanopattern with QDs, atmospheric plasma treatment was performed for 30 s. As the produced nanopatterned film was covered with hydroxyl groups, its surface was hydrophilic. Subsequently, green QDs were spin-coated from a toluene suspension for 1 min at 4000 rpm (see in Supplementary Figure S1). In the next step, the deposition of the 10 nm SiO_2_ layer and atmospheric plasma treatment were repeated to improve surface adhesion, followed by spin coating red QDs. The fabricated nanopatterned QD film consisted of sequential layers of green QDs, SiO_2_, and red QDs, counting from the bottom. Apart from this, to further enhance the efficiency, further QD and SiO_2_ layers were added to fabricate a double green/red QD layer structure used for characteristic analysis and application.

**Figure 2:**
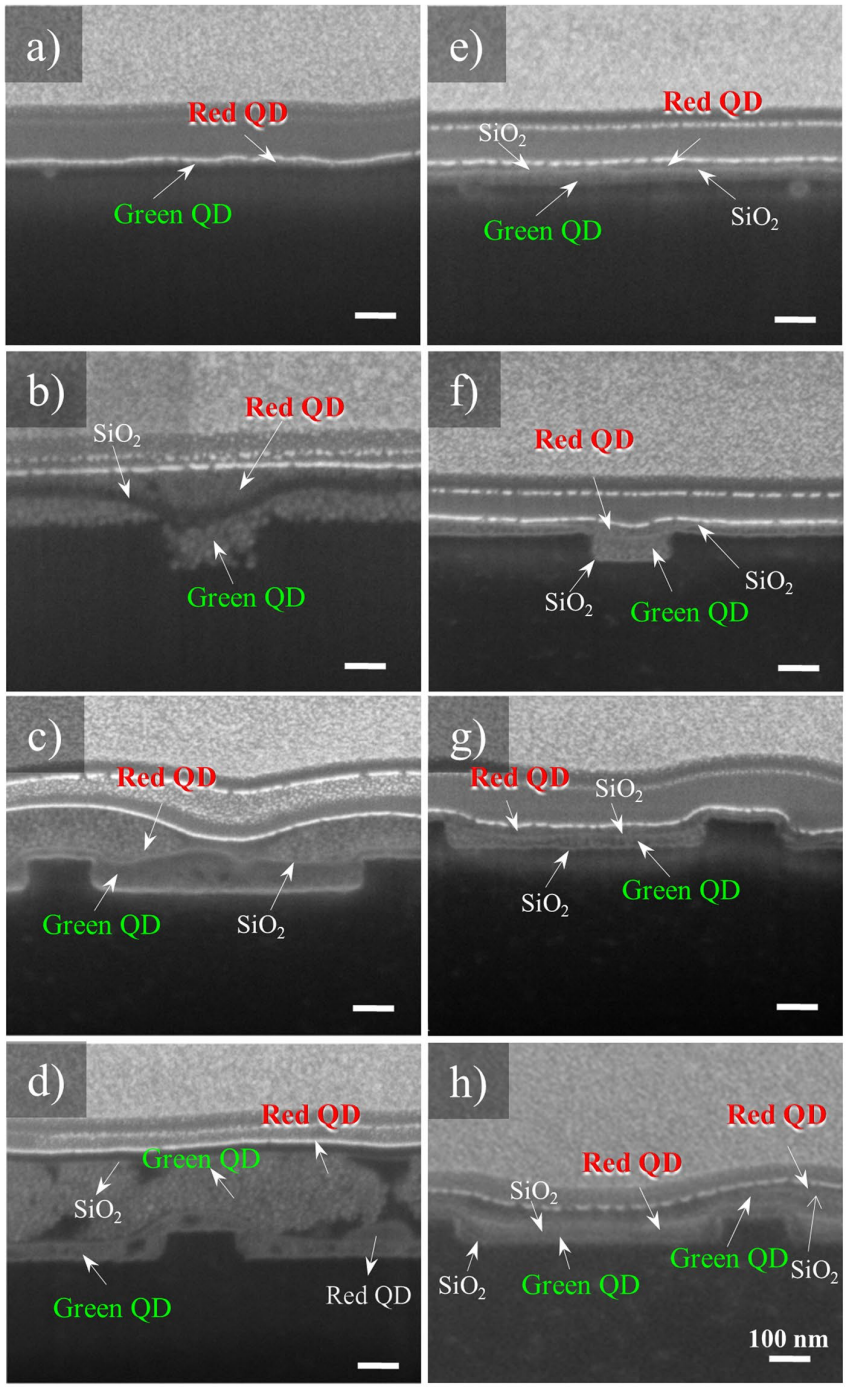
FIB image of the nanopatterned QD film layers. (**a**) Single QD layer obtained by spin coating on a flat PET film. (**b**) Single QD layer obtained by sequential deposition of green QDs, SiO_2_, and red QDs on a pattern with a line width of 200 nm and pitch of 800 nm. (**c**) Single QD layer obtained by sequential deposition of green QDs, SiO_2_, and red QDs on a pattern with a line width of 600 nm and pitch of 800 nm. (**d**) Double QD layer with additional sequential deposition of spin-coated layers of green QDs, SiO_2_, and red QDs on a pattern with a line width of 200 nm and pitch of 800 nm. (**e**) Single QD layer obtained by sequential deposition of red QDs, SiO_2_ (O_2_ plasma), and green QDs on a flat PET film, (**f**) Single QD layer obtained by sequential deposition of red QDs, SiO_2_ (O_2_ plasma), and green QDs on a pattern with a line width of 200 nm and pitch of 800 nm. (**g**) Single QD layer obtained by sequential deposition of green QDs, SiO_2_ (O_2_ plasma), and red QDs on a pattern with a line width of 600 nm and pitch of 800 nm. (**h**) Double QD layer with additional sequential deposition of green QDs, SiO_2_(O_2_ plasma), and red QDs on a pattern with a line width of 200 nm and pitch of 800 nm.

To examine the properties of the fabricated nanopatterned QD film, afocused ion beam (FIB; Helios NanoLab,FEI, Netherlands) technique was used for surface analysis. Figure 2 shows the Focus Ion Beam (FIB) analysis results for a layer section of the fabricated nanopatterned QD film. Figure 2a–d show the untreated nanopatterned QD film layer, and Fig. 2e–h show the nanopatterned QD film layer subjected to O_2_ plasma treatment. The analyzed pattern features a line width of 200 nm, pitch of 800 nm, and depth of 100 nm (Fig. 2b,f). When this pattern was duplicated to create another pattern in the reverse direction, a line width of 600 nm, pitch of 800 nm, and depth of 100 nm were obtained. For the duplicated nanopatterned film (Fig. 2c,g) with these dimensions, the double coating layer was fabricated as in Fig. 1j (Fig. 2d,h). As a result, similar to the film with an untreated QD layer surface (Fig. 2a–d), the filmfabricated by spin coating exhibited serious QD aggregation (Fig. 2b–d), with the QD layer coating being non-uniform because QDs were not filled in the pattern.

However, the nanopatterned film treated with O_2_ plasma to improve the QD filmdeposition was negatively charged, providing a hydrophilic surface.

In addition, during the plasma treatment, –OH groups are formed on the surface,which increasesthe particle adherence. In addition,XPS data shown in supplementary Figure S2 also reveals the higher intensity of the binding energy of the SiO_2_ layer^34^. Therefore, the deposition parameters were improved, resulting in better surface adhesion during spin coating and thereby enabling the pattern to be easily filled by the QD suspension.

As a result, a uniform QD layer was formed on the atmospheric plasma–treated patterned surface covered by SiO_2_ (Fig. 2e–h). In addition, a uniform 10 nm SiO_2_ layer was formed between the nanopatterned QD film surface and green and red QD layers, reducing QD aggregation, as manifested by the uniform coating observed in Fig. 2e–h.

**Figure 3:**
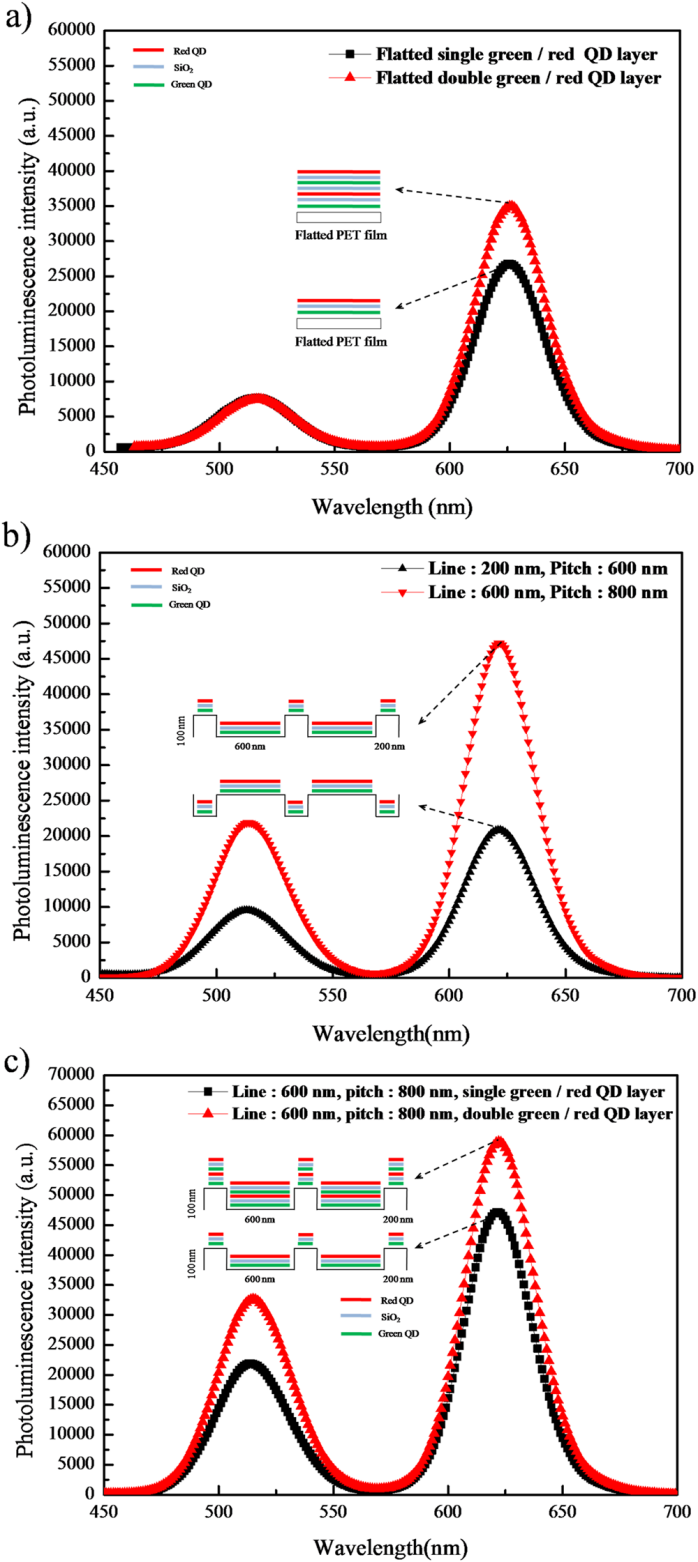
Experimental results obtained for nanopatterned QD films. (**a**) PL spectra of single green/red and double green/red QD layers on a flat PET film. (**b**) PL spectra of the line-patterned QD layer. (**c**) PL spectra of single green/red and double green/red QD layers on a nanopatterned PET film.

The characteristics of the fabricated nanopatterned QD films vary according to the nanopattern size and number of spin coating cycles, as evidenced by the photoluminescence (PL) spectra of these films. Figure 3a shows a Flatted single and double layer nanopatterned QD film subjected to FIB analysis (Fig. 2a,e), revealing that the highest intensities of the green and red peaks were obtained at 513 and 625 nm, respectively. The maximum PL intensity was observed in a Flatted double layer nanopatterned QD film with a large pattern size (Fig. 2e), and a relatively low PL intensity was observed in the film shown in single layer nanopatterned QD film (Fig. 2a). Fig. 3b is the result of PL properties of line width of 600 nm, pitch of 800 nm and depth of 100 nm (Fig. 2g) and PL properties of line width of 200 nm, pitch of 800 nm and depth of 100 nm (Fig. 2f). This can be rationalized by the fact that more QDs fill a larger nanopattern space, resulting in a two times higher PL intensity of the film (Fig. 2g), compared to Fig. 2f, for the pattern with a line width of 600 nm, pitch of 800 nm and depth of 100 nm in Fig. 3b.

Thus, the PL intensity of each QD layer could be adjusted, because the thickness and width of these layers can be tuned by varying the size and depth of the nanopattern. Thus, the color of red and green QDs used in this experiment can also be adjusted. To confirm the improvement in the nanopatterned QD film intensity, we attempted to characterize the multiple-deposited layer by measuring the PL propertiesof green and red QD films deposited as single layers and as a double layer on a nanopatterned QD film, where each QD layer was deposited twice. PL measurements (Fig. 3c) were conducted for nanopatterned QD films examined by FIB (Fig. 2g,h). Therefore, we deposited a larger number of QD layers on the fabricated nanopatterned film to improve its characteristics, achieving higher intensity and uniform coating. The double-layered nanopatterned QD film shows a 75% increase in the PL intensity for both green and red QDs, indicating considerable improvement. According to the size and depth of the nanopattern, the QD layer thickness could be adjusted in the range of dozens to hundreds of nm, and QD films with higher PL intensity could be fabricated by sequential deposition of additional layers over the double layer. If several QD layers are added, various colors could be realized, enabling full-color tuning and hologram applications, subject to blue color implementation.

The viewing angle is one of the important aspects, now being implemented in large-area displays, although it was not implementedbefore in transparent displays or is not found in existing displays. In addition to the display, many applications are required to provide accurate images from various angles to the observer. The developed nanopatterned QDs film can be used for both indoor and outdoor light interference using a laser light source. This is owing to the strong excitation of the QDsat certain wavelengths, and this property is very favorable in terms of the viewing angle (Supplementary Figure S5). However, since the laser source is a very intense light source, power control of the intensity is required (Tables S1, S2) and the location of the laser source needs to be altered such that it is not projected directly onto the eyes of the viewer (Supplementary Figure S6(d–f)). Therefore, in this study, the intensity was identified by controlling the viewing angle of the laser source on the nanopatterned QD film.

**Figure 4:**
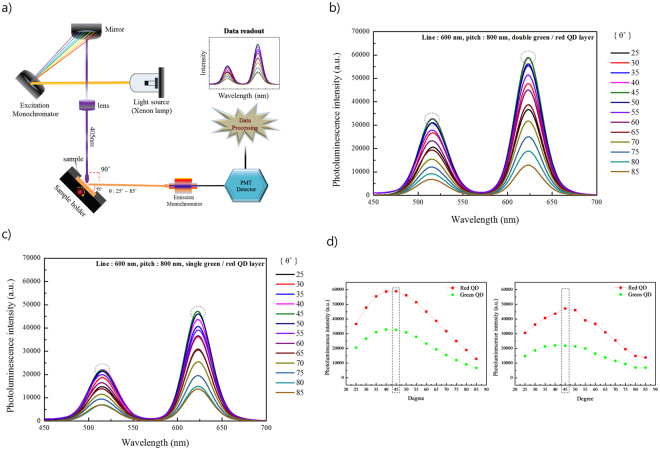
QD PL intensity as a function of the angle of incidence: (**a**) The measurement setup.PL intensity *vs*. incident angle for (**b**) a nanopatterned double layer film, and (**c**) a nanopatterned single layer film. (**d**) Intensity distribution of the PL of the nanopatterned double (**i**) and single (**ii**) QD films with respect to the angle of incidence *θ*.

Figure 4a shows the measured intensity of the nanopatterned QD film according to the angle of incidence of the laser, using the most efficient PL emission from Fig. 3b–c. In order to measure a wide range of incident angles, the nanopatterned QD film was placed in front a back plate capable of measuring 25 to 85°, and a multi-wavelength xenon lamp was set at a wavelength of 405 nm for excitation. The xenon lamp and PL detector were fixed at 90° with respect to each other and the angle of the back plate was adjusted. Subsequently, the light from the incident xenon lamp was directed on the nanopatterned film, and the QD PL was measured by the PL detector. The angle of incidence of the xenon light on the film shown in Fig. 4b is 25° to85°. When the θ value was 45°, the PL intensity of the double nanopatterned QD film was the highest. The PL intensitydecreased upon increasing or decreasing the θ value, and the same tendency was also observed for the single nanopatterned QD film shown in Fig. 4c. These results were obtained by fitting the peak values of the PL intensities measured according to the incident angles and their distributions are shown in Fig. 4d.

**Figure 5:**
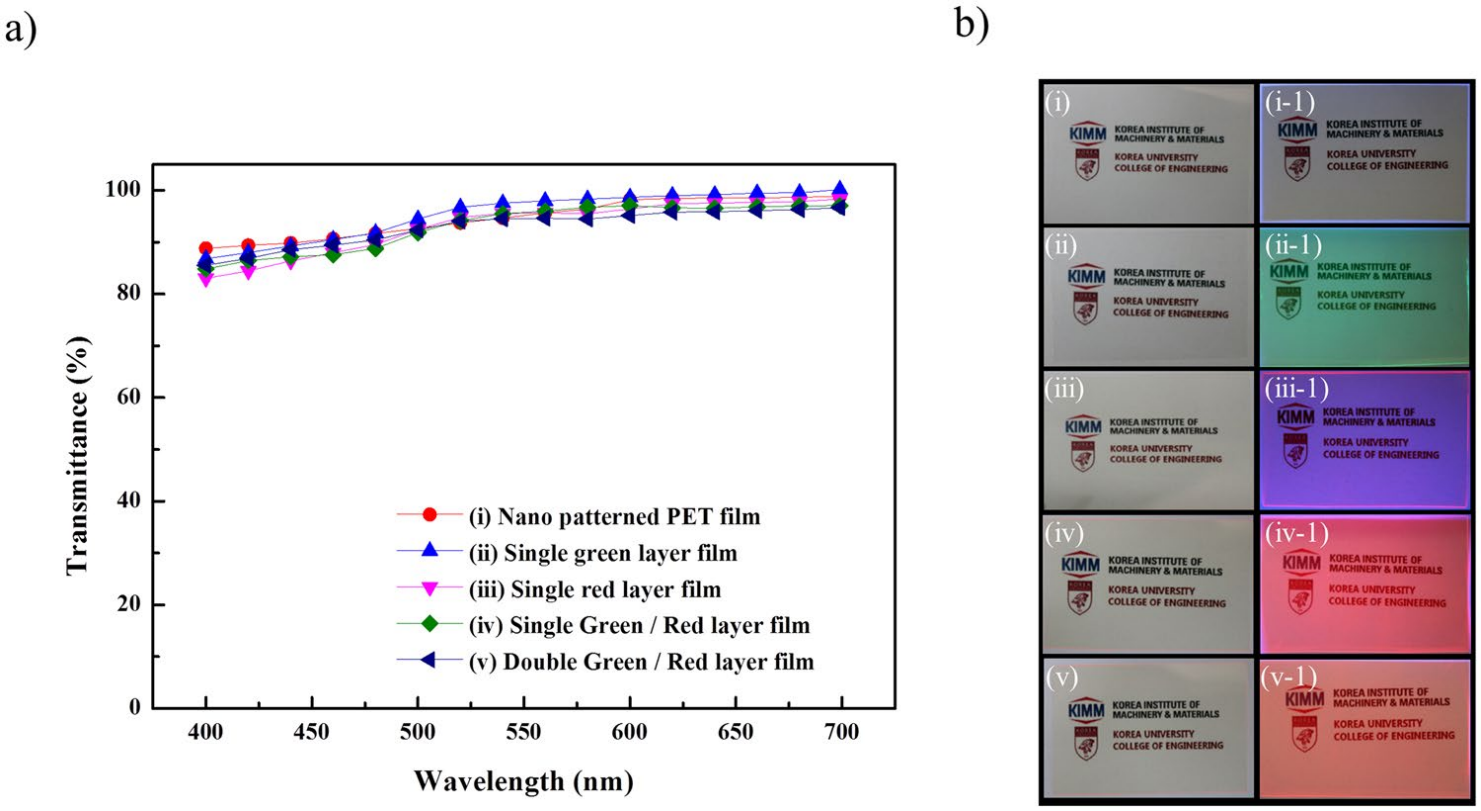
(**a**) Transmittances of the films. (**b**(i–v)) Transmitted images of the nanopatterned QD films used for the transmittance measurements. (**b**(i–1)–(v–1)) Transmitted images of a film with nanopatterned QDs exposed to 365 nm UV radiation.

Thus, multiple QD layers were spin-coated on the nanopatterned film fabricated by NIL to produce the nanopatterned QD film, whose transmittance was measured to verify its utility in transparent displays. Figure 5a displays the transmittance of each nanopatterned QD film fabricated by spin coating, with the transmittance of the PET film taken as the reference. Figure 5a(i–v) show the nanopatterned PET films with the pattern size and structure corresponding to those in Fig. 5b. Figure 5a(i) depicts a nanopatterned PET film with no deposited QDs, Fig. 5a(ii) shows a single green QD layer film, Fig. 5a(iii) displays a single red QD layer film, and Fig. 5a(iv–v) exhibit the single and double QD films characterized in Fig. 5b, respectively. Five types of films were examined in total. As a result, transmittances of 88 and >92% were observed at 400 and >500 nm, respectively (Fig. 5a(i)), with the corresponding values for Fig. 5a(ii),(iii) being equal to 80 and 95%. In Fig. 5a(iv), transmittances of 83 and >90% at 400 and >500 nm were observed, respectively. Finally, transmittances of 85 and >94% at 400 and >500 nm, respectively were observed in Fig. 5a(v). Thus, all the QD filmsshowed transmittances exceeding those of the existing transparent displays (40%) by more than a factor of two at all visible light wavelengths. Figure 5b shows a transmitted image of the measured film, and Fig. 5b(i–v) show the background image of the transmitted film. Figures 5b(i–1) to 5b(v–1) show films excited by UV light at 365 nm, revealing that the color as well as the background images could be observedin these films.

We used a blue LED with a wavelength of 450 nm and a blue laser source with a wavelength of 405 nm to generate the image on the fabricated nanopatterned QD film. However, the blue LED driven by the low current had a very low power output and its wavelength (450 nm) is higher than that of the laser (405 nm). Therefore, the excitation efficiency of the QD was low. It was also unsuitable as a source for creatingimages on the nanopatterned QD films because the LED light spreads widely (see in Supplementary Figure S3, Table S1). On the contrary, the laser source could create images on the nanopatterned QD film, but the power of the projected laser was very high, causing damage to the skin or eyes. Therefore, in this study, a module using a 1k ohm potentiometer was fabricated and a power output test was performed for the405 nm blue laser source (see in Supplementary Figure S3). Finally, to minimize damage to the human body, the experiment was performed using 225 mW laser power with 90 mA applied at 2.5 V (see in Supplementary Table S2).

**Figure 6:**
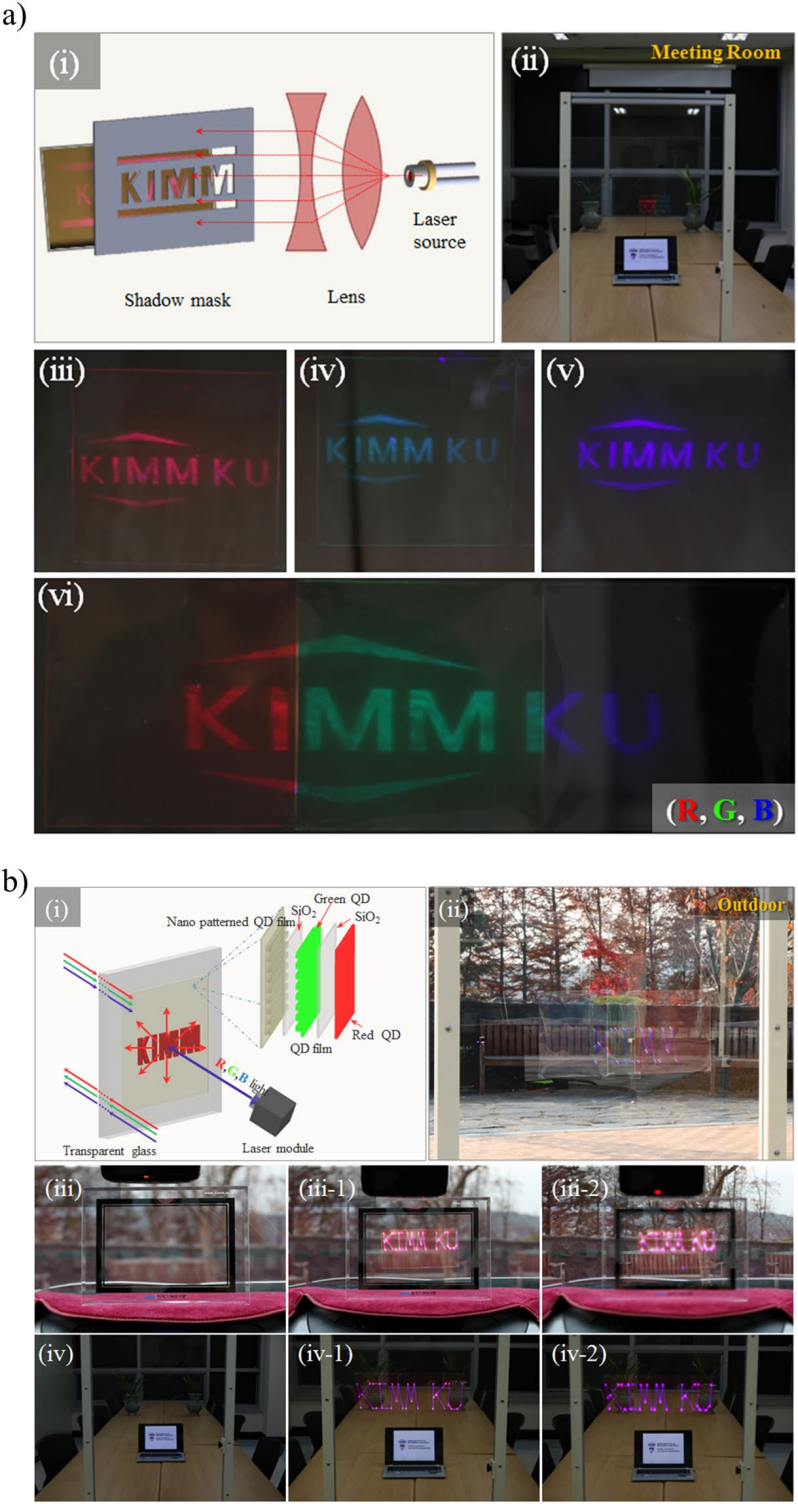
Demonstration of a transparent display. (**a**(i)) Implementation of a transparent display using a 405 nm laser light source and shadow mask: (**a**(ii)) “KIMM KU” image presented on transparent glass by a laser module in a meeting room, (**a**(iii–vi)) “KIMM KU” R,G, B image presented on transparent glass using a 405 nm laser source and shadow mask. (**b**(i)) Implementation of a transparent display using a laser module.“KIMM KU” image presented on transparent glass by the laser module:(**b**(ii)) outdoor, (**b**(iii)) inside a car, (**b**(iv)) inside a meeting room.

As shown in Fig. 6a(i), a “KIMM KU” image was projected onto the surface of a nanopatterned QD film attached to transparent glass using a laser module to examine the applicability of this film to transparent displays. Three 12 × 12 cm^2^ nanopatterned QD filmpieces were used for this experiment. As shown in Fig. 6a(ii), a shadow mask was used to create an image in front of the laser source, and an excited image was obtained on the nanopatterned QD film attached to transparent glass (see in Supplementary Figure S5). As for Fig. 6a(iii,iv,v) “KIMM KU”R (iii), G (iv), B (v) image presented respectively on transparent glass using a 405 nm laser source and shadow mask. And Fig. 6a(vi) which is 36 cm × 12 cm^2^ showed a large area image. As shown in Fig. 6b, exposure of the nanopatterned QD film to laser radiation resulted in QD excitation and projection of the KIMM KU image (Fig. 6b(i)). Figure 6b(i) is an experiment using laser module. Focusing the camera on the background situated 5 m behind produced a clear projection of the KIMM KU image in outdoor on the nanopatterned QD film (Fig. 6b(ii)). However, the laser light simply passed throughthe transparent glass with no nanopatterned QD film attached, and no image was projected (Fig. 6b(iii,iv)). Moreover, the experimental result shown in Fig. 6b reveals that the image is projected on the nanopatterned QD film attached on the transparent glass (Fig. 6b(iii–1, iv–1)), with the same result obtained by focusing the camera on the background situated 5 m behind (Fig. 6b(iii–2, iv–2)). Further, KIMM KU image on transparent glass inside a car using a laser module is also presented (see in Supplementary Figure S5). The experimental setup and related processes are described in detail in the Supplementary Information.

## Conclusions

In summary, a nanopatterned QD film for transparent displays was fabricated by spincoating QDs on a nanopatterned film. Additionally, two nanopattern types were formed on the QD film to improve its characteristics, corresponding to line widths of 200 and 600 nm with the pitch and depth being same at 800 and 100 nm. The QD coating thickness could be effectively increased by varying the line width and depth of the used pattern. Moreover, the QD filmcolor could be adjusted by repeated coating of the QD layers, and the problem associated with QD aggregation was overcome by depositing SiO_2_ layers between each QD layer and the nanopattern. The fabricated nanopatterned QD film showed an average transmittance of >80%, exceeding the transmittances of existing transparent displays (40%) more than two-fold. Tests in indoor and outdoor spaces revealed that any image could be realized, irrespective of the location and background light, even in places where interference of lightexisted. The produced film minimizes light loss, which is the main limitation of transparent displays, and can be implemented in various applications, representing a next-generation display technology that can enable the realization of any image, even in spaces exposed to visible light.

## Methods

### Materials

Core/shellCdSe/ZnS quantum dots manufactured by Uniam. Co., Ltd. were utilized in the form of a toluene suspension (10 mg/mL). The red-emitting CdSe inner core has a thickness of 4 nm, and the total diameter (including ZnS in the outer core) equals 10 ± 1 nm. The green-emitting CdSe inner core is 3 nm thick, with the total outer diameter being 10 ± 1 nm. PLmaxima for red and green QDs were observed at 513 and 625 nm, respectively. The nanopattern duplicated by the nanoline master was produced on a polyurethane series UV resin fabricated by combining OH and NCO monomers. For this, a UV resin, MINS-311RM (Minuta Co., Ltd.) was used. A 0.1 T PET film was used as a substrate owing to its good flexibility and transmittance.

### Nanoimprint lithography

The line master with several nanosizedline patterns used to fabricate the nanopatterned film was ultrasonicated in a Nano-Strip solution for 30 min and sequentially washed with acetone, ethanol, and isopropyl alcohol, followed by blow-drying with a nitrogen gun. The dried line master was subjected to 30 min anti-adhesion treatment in a furnace at 80 °C to obtain self-assembled monolayers for reducing the surface temperature during imprinting. The nanopatterned film fabricated by NIL was UV-cured for 90 s. After separating the duplicated nanopatterned film from the master, it was post-cured for 90 s. To prevent the aggregation of green and red QDs, a buffering 100 Å SiO_2_ layer was deposited using an E-beam evaporator. O_2_ plasma treatment was performed for 30 s to improve the surface energy of the quantum dots layer during spin coating, rendering the SiO_2_ layer negatively charged and hydrophilic.

### Device characterization

PL spectra of the nanopatterned QD films were recorded using a UV-Vis Fourier transform near infrared fluorescence spectrometer (FS-2, Scinco Co., Ltd.). A 5 × 5 mm^2^ area of the nanopatterned QD film was used for measurements, and QD emission was monitored every second. A Photo Multiplier Tube voltage of 450 V and an integration time of 20 ms were used. The values of the emission filter and excitation filter were setup by air condition, with the emission start and end measured at 450 and 720 nm, respectively. In addition, an analyzer-type UV-Vis spectrophotometer (S-3100, Scinco Co., Ltd.) was used to measure the transmittance. Transmittance of the 0.1-T PET film was first recorded as a reference, and then the transmittance of each sample film was analyzed. To realize images on the fabricated nanopatterned QD film, two types of lasers were used in addition to a laser module (wavelengths: red, 638 nm; green, 532 nm; blue, 445 nm and laserpower: red, 300 mW; green, 150 mW; blue, 600 mW), which can be applied for red, green, and blue colors, and a 405-nm laser source power (max: 500 mW, min: 130.5 mW)^35–39^.

## Electronic supplementary material

Supplementary Information
