# Supplementary material for: Transparent Displays Utilizing Nanopatterned Quantum Dot Films
Source: Sci Rep. 2018 Feb 6;8:2463. doi: 10.1038/s41598-018-20869-1 (PMC5802736; doi:10.1038/s41598-018-20869-1)
Supplement: Supplementary file 1 — Supplementary Information [file 41598_2018_20869_MOESM1_ESM.doc]

**Supplementary Information**

Transparent Displays Utilizing Nanopatterned Quantum Dot Films

Sang-ho Shin^1,2^, Boyeon Hwang^1^, Zhi-Jun Zhao^2^, So Hee Jeon^2^, JooYun Jung^2^, Ji-Hye Lee^2^, Byeong-Kwon Ju^1*^, and Jun-Ho Jeong^2*^

^1^Department of Electrical Engineering, College of Engineering, Korea University, Seoul 02841, Korea

^2^Department of Nano Manufacturing Technology, Korea Institute of Machinery and Materials (KIMM), Daejeon 34103, Korea

**Supplementary methods**

***R, G, B QD layer spin coating***

In order to confirm the homogeneous coating of the QD layer, a toluene-based quantum dot suspension was spin-coated on a silicon wafer and nanopatterned film. Homogeneous coating of the R, G, and B QD suspension on the silicon wafer was confirmed. To achieve homogenous coating on the nanopatterned film, SiO_2_ and O_2_ plasma treatment was performed on the film surface, resulting in a homogeneous coating.

***405 nm blue LED, 405 blue laser source output control***

To control the 405 nm blue laser source and blue LED used in this study, an electric circuit using a 1 kΩ potentiometer was constructed. 5 V was applied to the constructed circuit to drive the laser source, and a potentiometer was used to control the resistance. The voltage was measured using an oscilloscope and the maximum output was measured using a power meter. When voltage was applied and electricity flowed, the resistance was increased by 1 kΩ using the potentiometer, both the voltage and the corresponding output vary according to Ohm’s law. For the 405 nm blue laser source, a current of 87 mA resulted in 130.5 mW output at a minimum of 1.5 V and 500 mW output at a maximum of 4 V. Under the same conditions, the 405 nm blue LED provided 12 mW output at 12 mA for a minimum of 1 V and 56 mW of output at 20 mA for 2.8 V.

***PL intensity characteristics of a nanopatterned QD film based on the blue LED power***

Experiments were conducted to examine the effect of blue LED on the PL intensity of nanopatterned QD films. As shown in Figure S1C, a film was placed on LED and the variation in the PL intensity of the nanopatterned QD film with voltage was observed using a 1 kΩ potentiometer. As a result, the change in PL of the nanopatterned QD film was considerably small than the change in the PL intensity from the blue LED. Owing to the low output and light emitting characteristics of blue LED, the excitation characteristics of the QD film could not be maximized.

***Implementation of a transparent display***

Experiments were conducted in a meeting room to implement the nanopatterned QD film on transparent glass. To provide perspective, a notebook was placed 1 m from the screen and a flower pot was placed 3 m from the screen. With this setup in the office, the laser light was emitted to create images on the screen and these images were verified from various angles. In addition, we confirmed that the images could be displayed outdoors and in an automobile to demonstrate that it can be applied in various new applications.


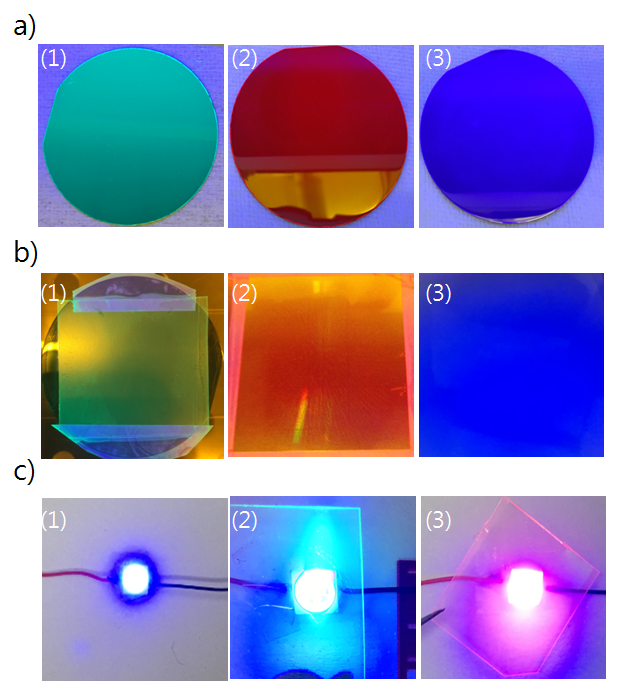


**Figure S1**. Nanopatterned films obtained by spin coating (a) (1) red, (2) green, (3) blue QDs on a Si wafer; (b) (1) red, (2) green, and (3) blue QDs on a nanopatterned film; (c) (1) Image showing the 405 nm blue LED light emission, (2) light emission image of a nanopatterned green QD film illuminated by a 405 nm blue LED, (3) light emission image of a nanopatterned red QD film illuminated by a 450 nm blue LED.


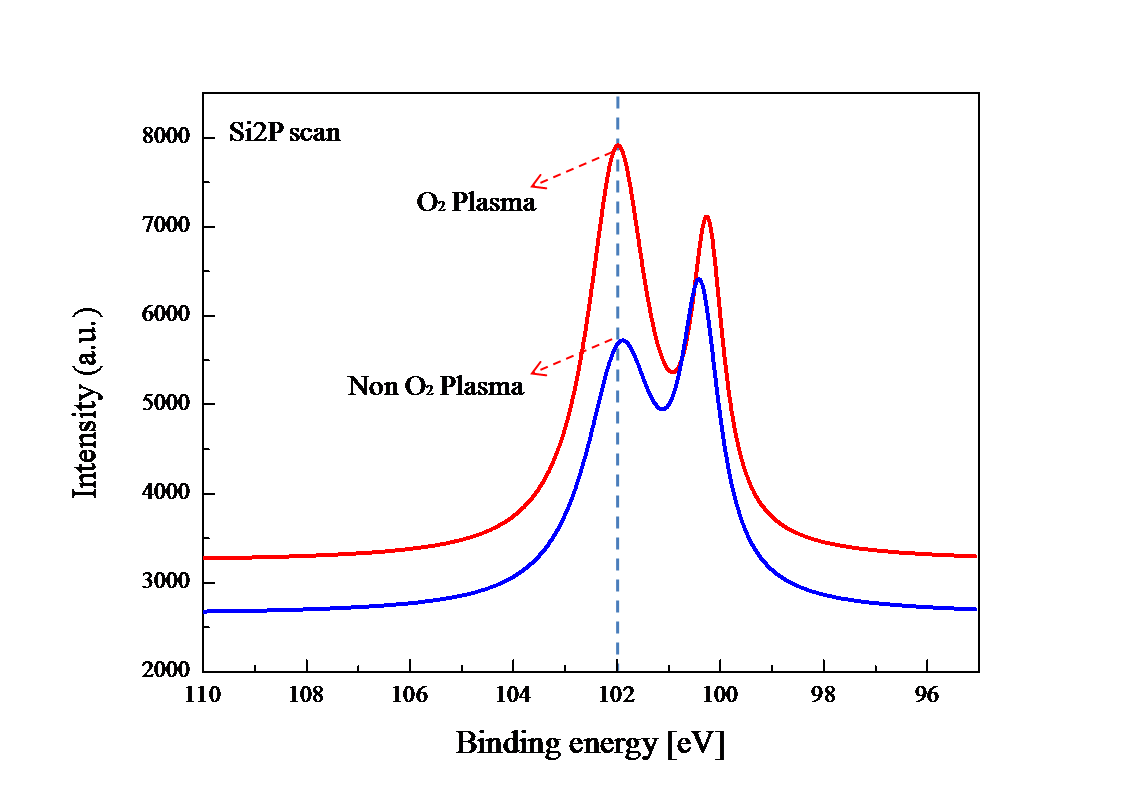


**Figure S2**. X-ray photoelectron spectra of various SiO2 surfaces. Binding energy Intensity difference due to plasma treatment O_2_ plasma and non O_2_ plasma


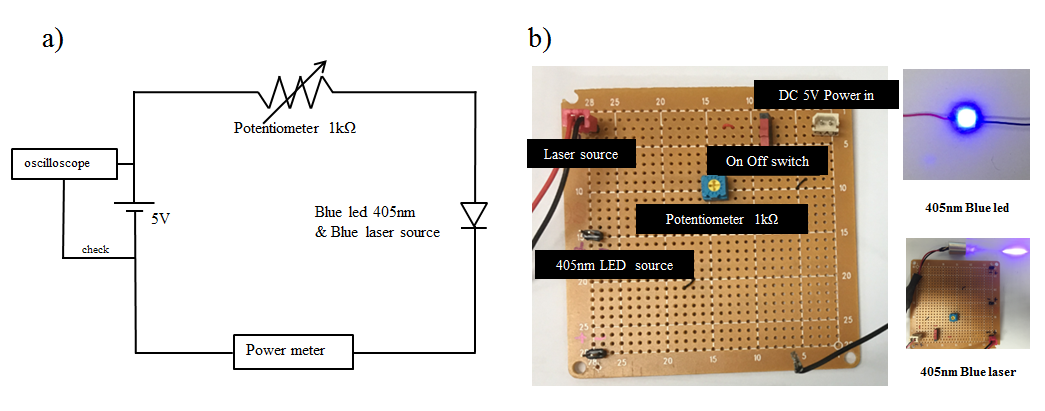


**Figure S3**. Quantitative output control method for 450 nm LED and a laser source. (a) An output control circuit diagram using a potentiometer. (b) A final output control board.


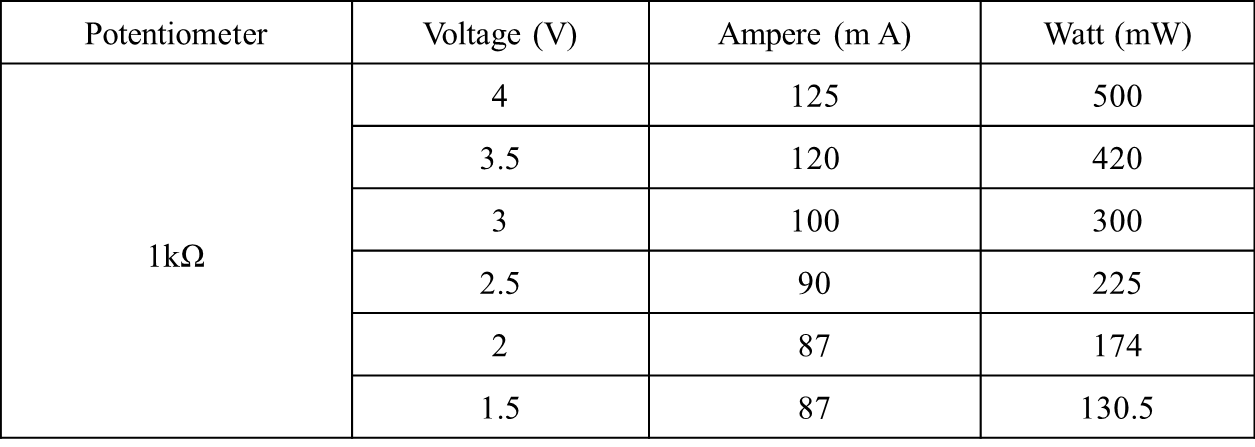


**Table S1**. Power output as a function of the voltage for a 405 nm blue laser source


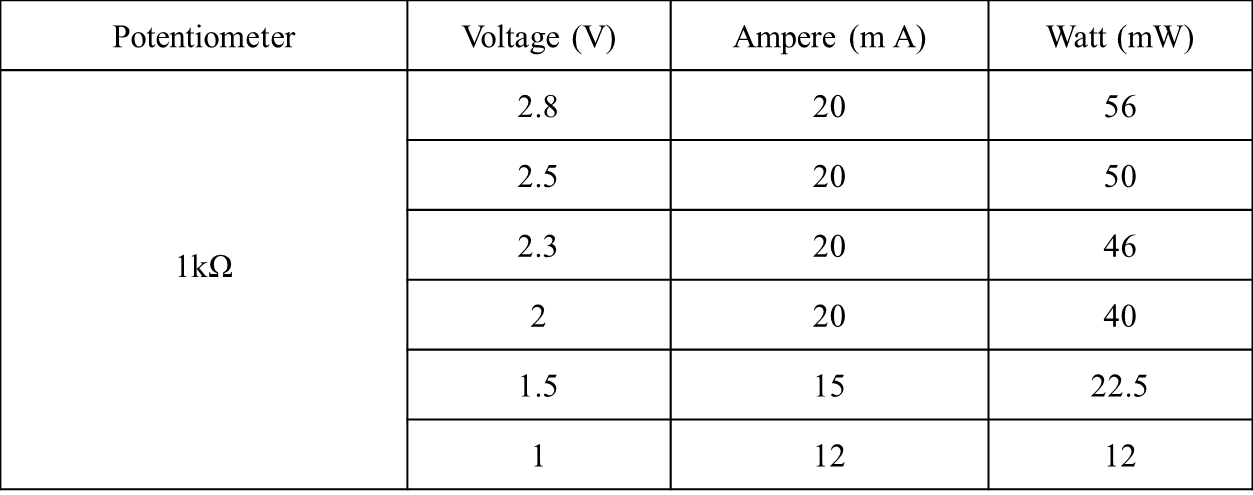


**Table S2**. Power output as a function of the voltage for 450 nm blue LED


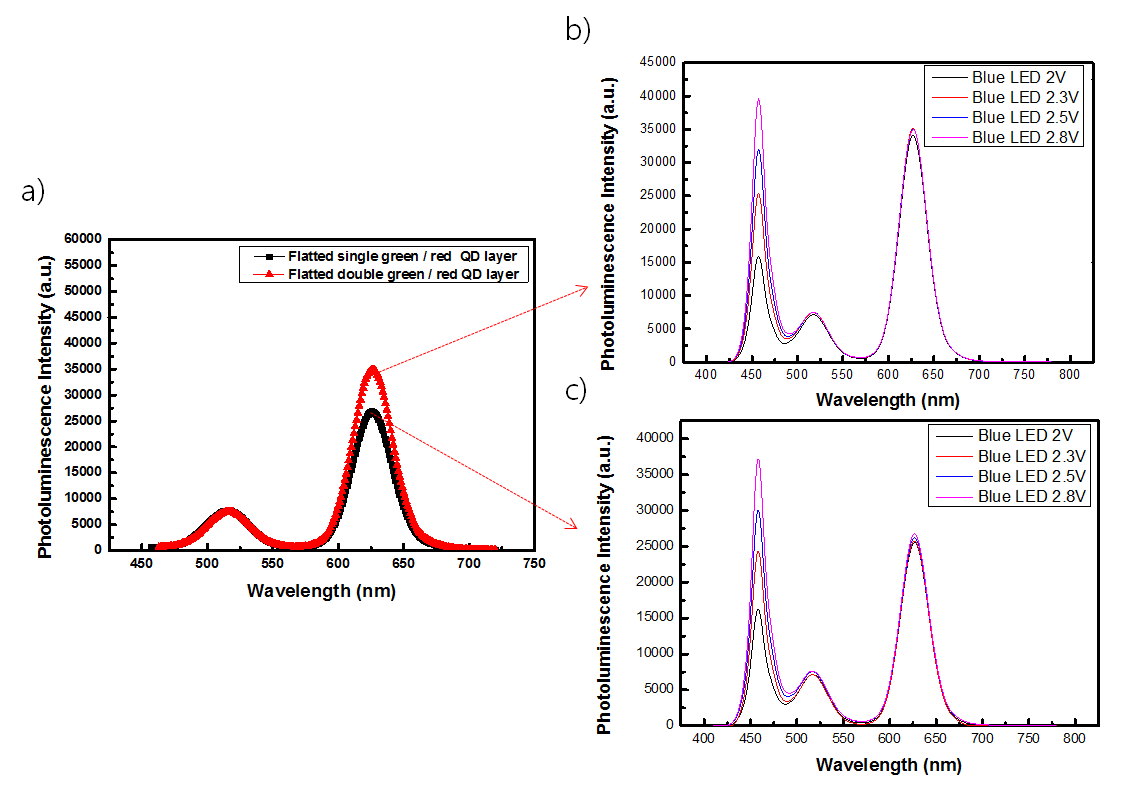


**Figure S4**. Variation of the QD PL intensity as a function of the blue LED power (450 nm) (a) PL intensities of a flat single green/red QD layer and flat double green layer, (b) PL intensity with respect to the voltage power of blue LED (450 nm) and PL intensity of the flat single green/red QD layer. (c) PL intensity with respect to the voltage power of blue LED (405 nm) and PL intensity of the flat single green/red QD layer.


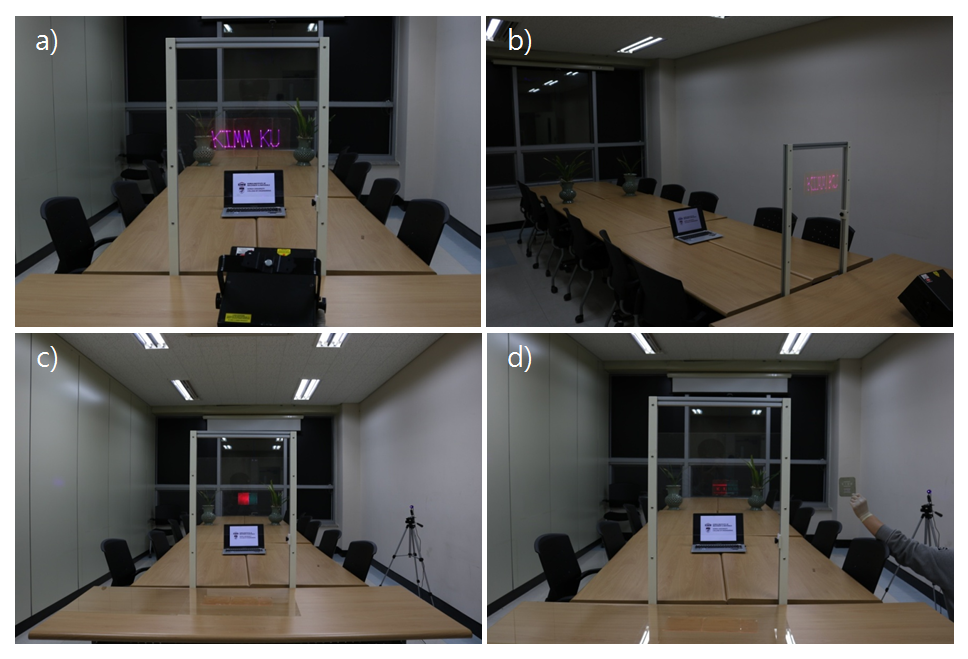


**Figure S5**. Design and implementation of a laser module for image projecting. (a) KIMM KU front view of the image and the laser module configuration. (b) Side view and module configuration of the KIMM KU image. (c) Implementation of a projecting image using shadow mask laser module alignment. (d) Implementation of KIMM KU image using a shadow mask.


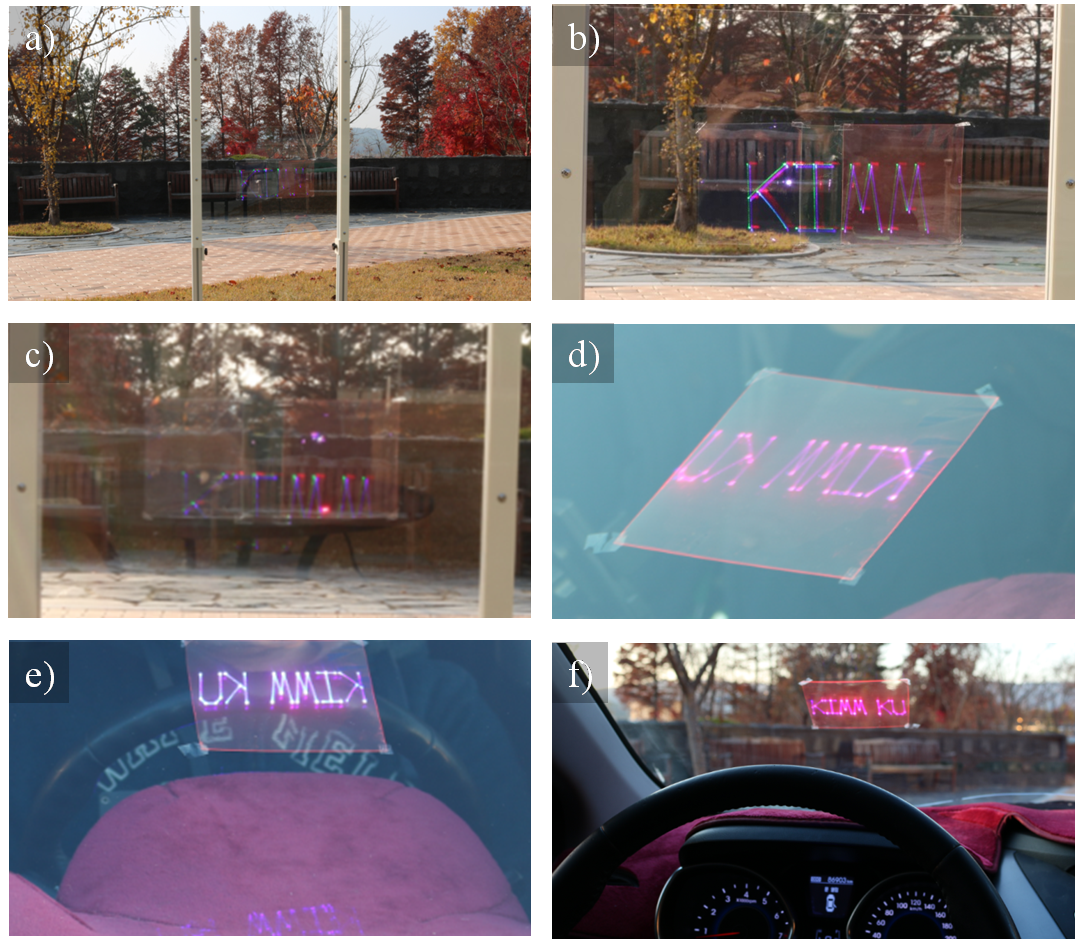


**Figure S6**. Future applications of the transparent display concept. (a–c) Transparent outdoor images. (d–f) Concept images applied in a car interior.

**References**:

[1] Kim, T. H., Cho, K. S., Lee, E. K., Lee, S. J., Chae, J., Kim, J. W., Kim, D. H., Kwon, J. Y., Amaratunga, G., Lee, S. Y., Choi, B. L., Kuk, Y.; Kim, J. M., Kim, K. Full-Color Quantum Dot Displays Fabricated by Transfer Printing. Nature photonics 5, 176–182 (2011).

[2] Uoyama, H., Goushi, K., Shizu, K., Nomura, H., & Adachi, C. Highly Efficient Organic Light-Emitting Diodes From Delayed Fluorescence. Nature, 492(7428) 234 (2012).
